# Scaling laws of plume-induced granular cratering

**DOI:** 10.1093/pnasnexus/pgad300

**Published:** 2023-09-20

**Authors:** Matthew T Gorman, Juan Sebastian Rubio, Miguel X Diaz-Lopez, Wesley A Chambers, Ashley M Korzun, Jason Rabinovitch, Rui Ni

**Affiliations:** Department of Mechanical Engineering, Johns Hopkins University, Baltimore, MD 21218, USA; Department of Mechanical Engineering, Johns Hopkins University, Baltimore, MD 21218, USA; Department of Mechanical Engineering, Johns Hopkins University, Baltimore, MD 21218, USA; NASA Marshall Space Flight Center, Huntsville, AL 35802, USA; NASA Langley Research Center, Hampton, VA 23666, USA; Department of Mechanical Engineering, Stevens Institute of Technology, Hoboken, NJ 07030, USA; Department of Mechanical Engineering, Johns Hopkins University, Baltimore, MD 21218, USA

**Keywords:** plume-induced cratering, supersonic jet

## Abstract

Extraterrestrial landing often requires firing a high-speed plume towards a planetary surface, and the resulting gas–granular interactions pose potential hazards to the lander. To investigate these jet-induced cratering dynamics, an experiment campaign covering a range of gas and granular properties relevant to the lunar and Martian environments was conducted in a large-scale vacuum chamber. Despite the variations in jet Mach number, mass flow rate, and composition of the granular phase investigated in this work, the observed time evolution of crater depth displays a consistent transition from an early-stage linear to a late-stage sublinear growth. To explain these scaling relations, a model that relates the kinetic energy gained by the particles per unit time to the power of the impinging jet is introduced. From this model, erosion rates and the critical depth at which the transition occurs can be extracted, and they are shown to depend on the gas impingement pressure, which was varied by changing ambient pressure, jet Mach number, mass flow rate, and nozzle height above the surface. These results highlight key mechanisms at work in the dynamics of plume-induced cratering and help to develop an understanding of optimal rocket engine firing times for future landings.

**Significance Statement**

Extraterrestrial bodies are covered with craters induced by asteroid impacts. In addition to those driven by natural processes, craters can also be generated by landing spacecrafts. This process can produce exciting new discoveries, e.g. the subsurface water on Mars was first exposed due to the explosive erosion during the Phoenix landing. Despite the clear distinction between cratering processes driven by natural asteroid collisions and man-made retropropulsion systems, the underlying physics related to the energetic intrusion into the granular phase have some remarkable similarities that allow us to model the process by making an analogy between the two seemingly different processes. The proposed model helps to explain the complicated supersonic plume-induced cratering process that was not clearly understood before.

## Introduction

**Fig. 1.**
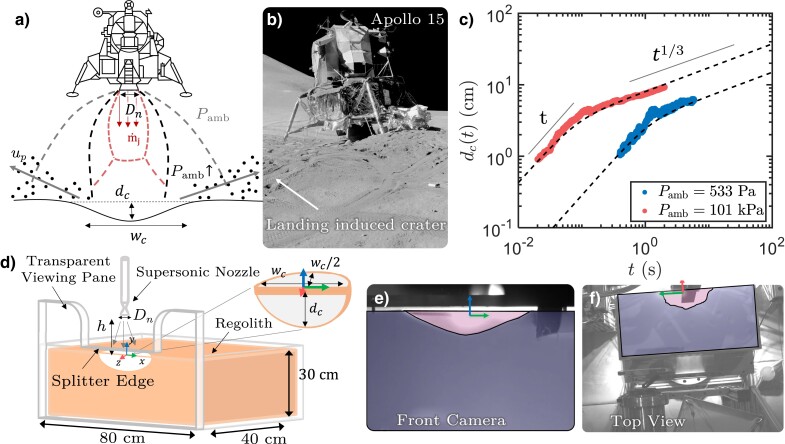
a) Schematic of PIC during an extraterrestrial landing. b) Picture of Apollo 15’s lunar module Falcon at rest on the rim of a 6-m wide crater (photo credit: NASA). The crater places Falcon at an 11-degree tilt. c) Sample data showing the evolution of the crater depth ($d_{c}$) over time *t* at two different pressures. (d) Schematic of the experimental setup in a large-scale vacuum chamber. e, f) Sample crater images taken by two high-speed cameras.

The impingement of a fluid jet on a granular surface, and the resulting plume-induced cratering (PIC), is important to a wide range of applications, from an octopus or a robot expelling a jet to burrow (1, 2) to the thruster from the Apollo Lunar Module eroding the lunar surface (as illustrated in Fig. 1a) (3). Prior studies of PIC have been conducted studying potential hazards to the Apollo lunar landings (4, 5) and the erosion of sand beds near hydraulic structures (6–9). Driven by the renewed interest in space exploration, planetary PIC and its associated erosion processes have received more attention recently (10–15). While there are many hazards caused by rocket-exhaust plumes, such as reduced visibility, vehicle damage, and the creation of uneven landing surfaces (the Apollo 15 lander experienced an 11-degree tilt in Fig. 1b), landing site erosion can also sometimes yield unexpected benefits, such as exposing water ice during the Mars Phoenix landing, which provided evidence for liquid saline water on Mars (16).

Several interaction mechanisms based on the importance of gas pressure and shear stress relative to the granular pore and lithostatic pressures have been proposed (13, 14, 17, 18). Experimental investigations of these mechanisms must deal with a large parameter space, as shown in Fig. 1a. The influence of the nozzle exit diameter ($D_{n}$) (19), nozzle height (*h*) (6), mass flow rate ($\dot{m}$) (12), particle parameters (shape, size, density) (20), and gravity (21) have all been studied for subsonic impinging jets. Given the experimental challenges, very few studies have been able to focus on the supersonic jet impingement, even though the jet Mach number could drastically change the jet structures, shock dynamics, and the interaction between gas and granular beds. In particular, it has been shown that complicated shock structures, including a sequence of Mach disks at the jet centerline and a large bow shock above the impingement surface, can develop for a supersonic jet (22–24). These introduced effects represent a new regime of the PIC problem that has not been adequately investigated previously. Pulsing the jet further complicates the problem and can lead to explosive erosion (25). However, the conditions under which a given interaction mechanism dominates remain unclear (14, 26).

PIC bears a certain resemblance to impact cratering, where projectiles, with diameters ranging from laboratory scales (cm) to solar system scales (10 km), impact on granular media and produce craters and ejecta (27–30). Impact cratering often involves additional complexities, such as shockwave heating of asteroid impactors (31) or viscous dissipation of liquid drop impactors (32). Nevertheless, an energy partitioning method that relates the kinetic energy of the impactor to excavated particles and heat loss [asteroids (27)] or surface energy and viscous dissipation [drops (32)] has achieved success in explaining the scaling laws between crater geometry and impactor energy.

In this article, experiments from a new campaign to study PIC in lunar and Martian environments are reported. Details of the set of experiments included in this analysis are provided in the Materials and methods section. To explain the time evolution of PIC, a power-balance framework inspired by the energy-based model from impact cratering is developed. The model captures the early- and late-stage active crater growth dynamics before the crater depth asymptotes to a steady-state value in the infinite time limit. This behavior is evident not only for many experiments conducted in this campaign but also for prior experiments in subsonic and terrestrial environments, which suggests a consistent behavior of PIC across a wide range of parameters.

## Challenges and solutions

To reproduce relevant low-pressure environments present on the lunar and Martian surfaces, our primary test campaign was conducted in a large cylindrical vacuum chamber (4.6 m D × 4.6 m H) at NASA Marshall Space Flight Center. Pressure rise during reduced atmosphere testing was mitigated by the volume of the chamber and, when possible, by running the chamber’s two 7.5 kW vacuum pumps during the test. The granular media used for this test campaign included monodispersed sand, glass beads, a bidispersed simulant mixture, and a tridispersed simulant mixture to represent lunar regolith and incrementally increase the complexity of the soil (for details, see Materials and methods section). Additional tests conducted at Johns Hopkins University (JHU) under Earth atmospheric pressure conditions were also included in this analysis.

The jet mass flow rate, $\dot{m}$, ranged from 0.32 to 8.6 g/s. The nozzle was placed at four different nozzle heights (*h*), normalized by the nozzle diameter ($D_{n}$), from 3 to 21.2. Experiments conducted in the vacuum chamber used a nozzle with a jet Mach number ($\mathrm{Ma}=u_{0}/c$, where $u_{0}$ represents the jet velocity and *c* indicates the speed of sound) of 5.3, while JHU tests were conducted with a sonic (Ma=1) jet.

The plate was aligned with the nozzle centerline to bisect the jet and expose the crater profile to the front camera. The soil bin was sufficiently deep (30 cm) to minimize the effect of the bottom boundary (the maximum crater depth for all datasets included in this analysis was 21.5 cm, or 72% of the soil bin depth). Most of the data presented in this work was obtained from the front-view camera (Fig. 1e). Three more cameras were positioned above the soil bin (Fig. 1f) to provide additional views of the crater geometry. Typical images captured by the front-view and top-view cameras are shown in Fig. 1e and f, respectively.

In Fig. 1e, the boundary of the crater was extracted based on the contrast of the image. Despite some visible dark shadows within the excavated region cast from the crater rim on the other side, a single threshold intensity value can still be defined to distinguish the excavated region (magenta area) from the undisturbed soil below. The depth $d_{c}(t)$ is measured from the undisturbed soil height to the lowest point on the profile, and the width $w_{c}(t)$ is defined as the largest horizontal dimension of the crater profile as measured from the front camera. Throughout this work, $w_{c}(t)/2$ is assumed to provide a good approximation of the average crater radius.

Figure 1c shows the time evolution of $d_{c}$ for two different cases at terrestrial (100 kPa, 1 atm) and near-Martian (0.6 kPa, 0.006 atm) pressure environments. For lower $P_{\mathrm{amb}}$, the jet expands further as it exits the nozzle in order to match the lower ambient pressure, which results in a lower erosion rate. Despite these variations in environmental conditions, these two curves share a similar trend: a linear growth stage followed by a transition to sublinear growth.

## Model

For craters produced by asteroid impacts, the Schmidt–Holsapple (S–H) scaling law was established by linking the crater size to the kinetic energy of the impactor (27). Here, a model for PIC analogous to the S–H scaling law is developed by also linking $d_{c}$ to the kinetic energy of an impinging jet. Unlike a solid impactor, an impinging jet does not have a discrete kinetic energy; instead, the jet continuously imparts energy to the granular medium. We assume the power of the jet remains constant throughout the PIC process. The total power scales with $\Delta Pu_{0}(\pi D_{n}^{2}/4)$, where ΔP is the characteristic pressure difference in this problem. While some of the impinging gas is directed away from the surface or permeates through the soil, the jet also imparts kinetic energy ($k_{p}$) to the excavated particles, which are accelerated to the ejecta velocity $u_{p}$ as they exit the crater.

Assuming that the particle kinetic energy gained per unit time ($dk_{p}/dt$) scales with the injected gas power yields the following expression:


(1) $$\Delta P\frac{\pi D_{n}^{2}}{4}u_{0}∼\rho_{p}\frac{d(w_{c}^{2}d_{c})}{dt}u_{p}^{2},$$

where $\rho_{p}$ and $u_{p}$ represent the particle density and velocity, respectively. $u_{p}$ is assumed to be a constant over time for a given experimental configuration. To quantify the time evolution of $u_{p}$ and validate this assumption, a separate high-speed camera was used throughout the test campaign to track ejecta velocity. Results from this camera suggest that, after a short initial transient period, $u_{p}$ indeed does not change significantly with time.

**Fig. 2.**
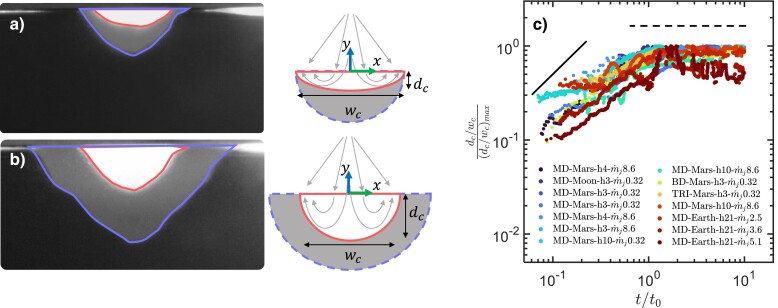
a, b) Overlaid experimental crater images accompanied by schematics of the crater evolution at a) t=0.5 s (red inner interface) and t=1.6 s (blue outer interface) and b) t=1.6 s (red inner interface) and t=9 s (blue outer interface); c) Crater aspect ratio ($d_{c}/w_{c}$) as a function of the normalized time. The dashed line represents a plateau, and the solid line represents linear relationship. The details of the legend can be found in the Materials and methods section.

Snapshots of the crater profile at two different times are overlaid in Fig. 2a and b to show the early-stage and later stage crater development, respectively. Early-stage crater evolution is directed primarily along the vertical direction with relatively insignificant growth of the width, i.e. $d[d_{c}(t)]/d[t]\gg d[w_{c}(t)]/d[t]$. This preferential erosion direction can be explained by the elevated surface pressure: when the jet first touches an unaltered surface, the pressure is applied normal to the flat surface and mainly pushes the crater down. The resulting growth follows a linear relationship with time.


(2) $$d_{c}(t)∼t\left(\text{Early Stage},\frac{\text{d}[d_{c}(t)]}{\text{d}t}\gg \frac{\text{d}[w_{c}(t)]}{\text{d}t}\right).$$

At a later stage, the crater growth in the vertical direction slows down. The gas pressure is still applied normal to the crater interface, but now that the interface is curved, the pressure force has an appreciable projection along the horizontal direction that serves to widen the crater. The outcome is shown in Fig. 2b; the overlaid images suggest that the crater width growth is proportional to the crater depth growth. As a result, later stage crater evolution follows a 1/3 power law.


(3) $$d_{c}(t)∼t^{1/3}\left(\text{Late Stage},\frac{\text{d}[d_{c}(t)]}{\text{d}t}\approx \frac{\text{d}[w_{c}(t)]}{\text{d}t}\right).$$

The critical transition time between the early and late stages is denoted as $t_{0}$, which will be used to separate the linear and sublinear regimes.

In addition to the qualitative pictures in Fig. 2a and b, we also show the ratio between depth and width, i.e. $d_{c}(t)/w_{c}(t)$ over time, for different types of granular and gas conditions. This ratio does not start from infinity; the finite area of the jet profile’s initial imprint on the granular phase produces a finite crater width at t=0. Since this ratio is normalized with its peak value, $d_{c}(t)/w_{c}(t)$ for all cases eventually plateaus to one. Note that both depth and width continue to increase throughout each test. An increase in $d_{c}(t)/w_{c}(t)$ indicates a faster growth rate in depth than in width, whereas a plateau of $d_{c}(t)/w_{c}(t)$ suggests a similar growth rate in all dimensions. In early times, the ratio clearly increases for all cases. Note that, if the increase follows a linear relationship as shown by the solid line, it would imply that the width is kept at a constant value and the relationship $d[d_{c}(t)]/dt\gg d[w_{c}(t)]/dt$ assumed in the model can be satisfied since $d[w_{c}(t)]/dt$ is zero. Although some cases do deviate from the linear relationship given the scatter of the data, the growth of $d_{c}(t)/w_{c}(t)$ over time still satisfies the assumption. In late times, a plateau can be observed for nearly all cases, suggesting a similar growth rate for both depth and width, again agreeing with the key assumption made in the model.

Prefactors are designated for each scaling law: $d_{c}(t)=f(\xi ,\eta )t$ and $d_{c}(t)=g(\xi ,\eta )t^{1/3}$. These two prefactors measure the erosion rates in the early and later stages, respectively. Both erosion rates depend on two variables, *ξ* and *η*, each of which represents a collection of parameters: *ξ* includes all the granular-phase properties, such as the particle size and density, interparticle cohesion, and soil compaction, while *η* includes all the gas-phase properties, such as the Mach number, flow rate, gas density, nozzle height, nozzle width, ambient pressure, and the jet pressure.

Although the two erosion rates depend on many parameters of both the granular and gas phases, there are two important predictions based on our power-balance model: (1) *ξ* and *η* affect the values of the erosion rates but not the scaling laws in the crater time evolution. Such scaling laws could work across a wide range of soil types and gas conditions. (2) f(ξ,η) and g(ξ,η) are not independent; rather, g(ξ,η) scales with $f^{1/3}(\xi ,\eta )$ based on Eq. 1 if all the variables apart from $d_{c}w_{c}^{2}$ remain constant over time.

To confirm these two predictions, the critical time $t_{0}$, extracted from Fig. 2c based on when the plateau is reached, is used to separate the time evolution of $d_{c}(t)$ into the linear and sublinear regimes, which in turn can be used to determine the respective erosion rates f(ξ,η) and g(ξ,η).

**Fig. 3.**
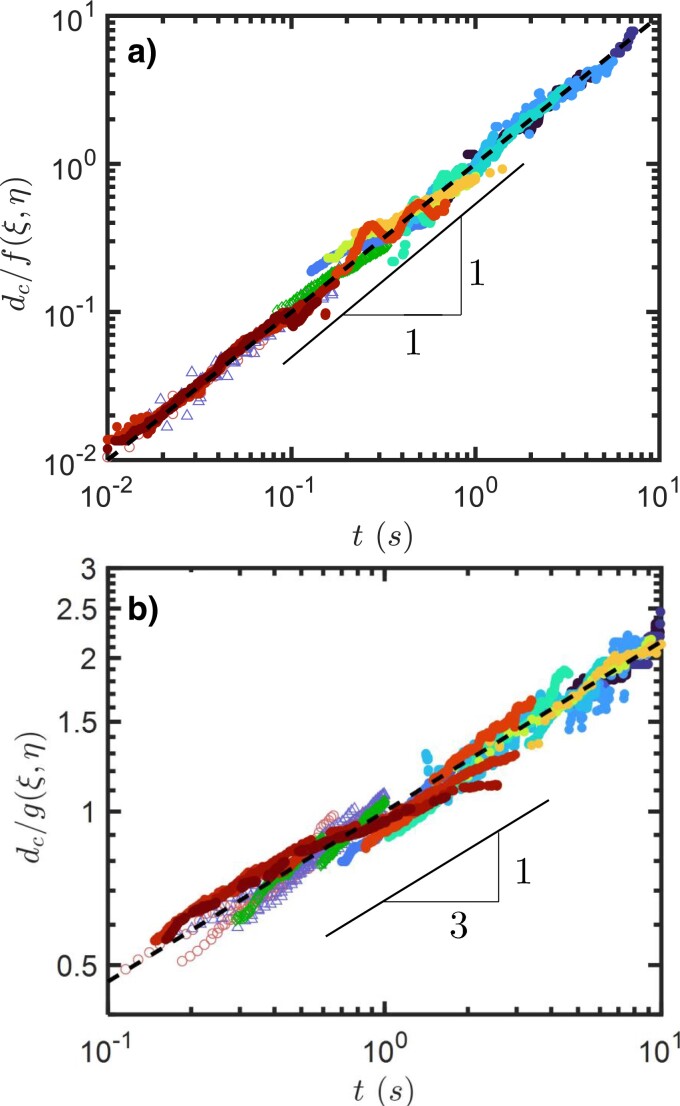
a) The normalized crater depth versus time for $t<t_{0}$. b) The normalized crater depth versus time for $t>t_{0}$. Filled markers with different colors are the same as those in Fig. 2c. Additional data from previous works, including (20) (circles), (33) (triangles), and (14) (diamonds) have also been added.

To isolate the relationship between $d_{c}$ and *t*, each regime, normalized by its respective erosion rate, is shown in Fig. 3. Within the range of parameters examined, the time evolution of the crater depth collapses onto a line for the early stage and onto a $t^{1/3}$ power law for late stage, extending over two decades for each regime. This finding demonstrates a consistency in time evolution independent of the jet and granular properties: the latter affect erosion rates, but not scaling laws with time.

**Fig. 4.**
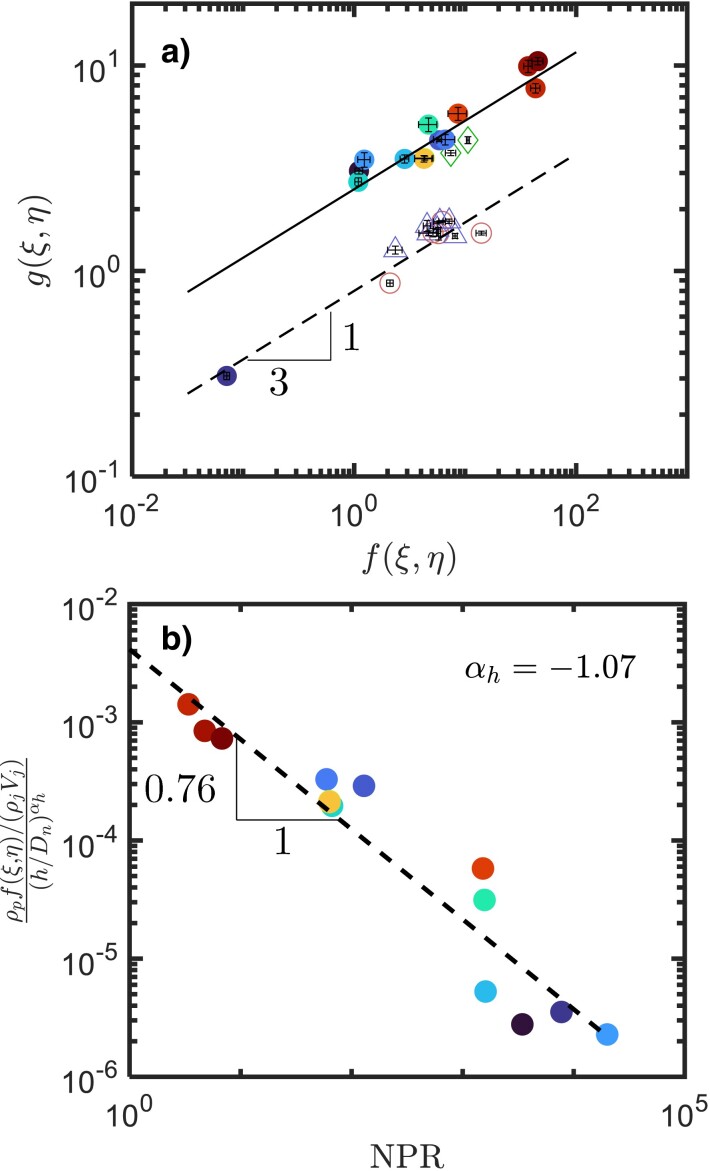
a) The late-stage erosion rate g(ξ,η) as a function of the early-stage erosion rate f(ξ,η). The lines indicate a 1/3 power law relationship. The symbols are consistent with those in Fig. 2c. Additional data from previous works, including (20) (circles), (33) (triangles), and (14) (diamonds) have also been added. The solid line passes through the sonic and supersonic cases, and the dotted line passes through the subsonic cases. b) The nondimensionalized erosion rate f(ξ,η) versus the nozzle pressure ratio (NPR).

To probe the second prediction from the model, f(ξ,η) and g(ξ,η) are plotted against each other in Fig. 4a. Given the parameters examined in this campaign, the linear erosion rate f(ξ,η) spans over three decades. The lines indicate the 1/3 scaling law predicted by the model.

**Fig. 5.**
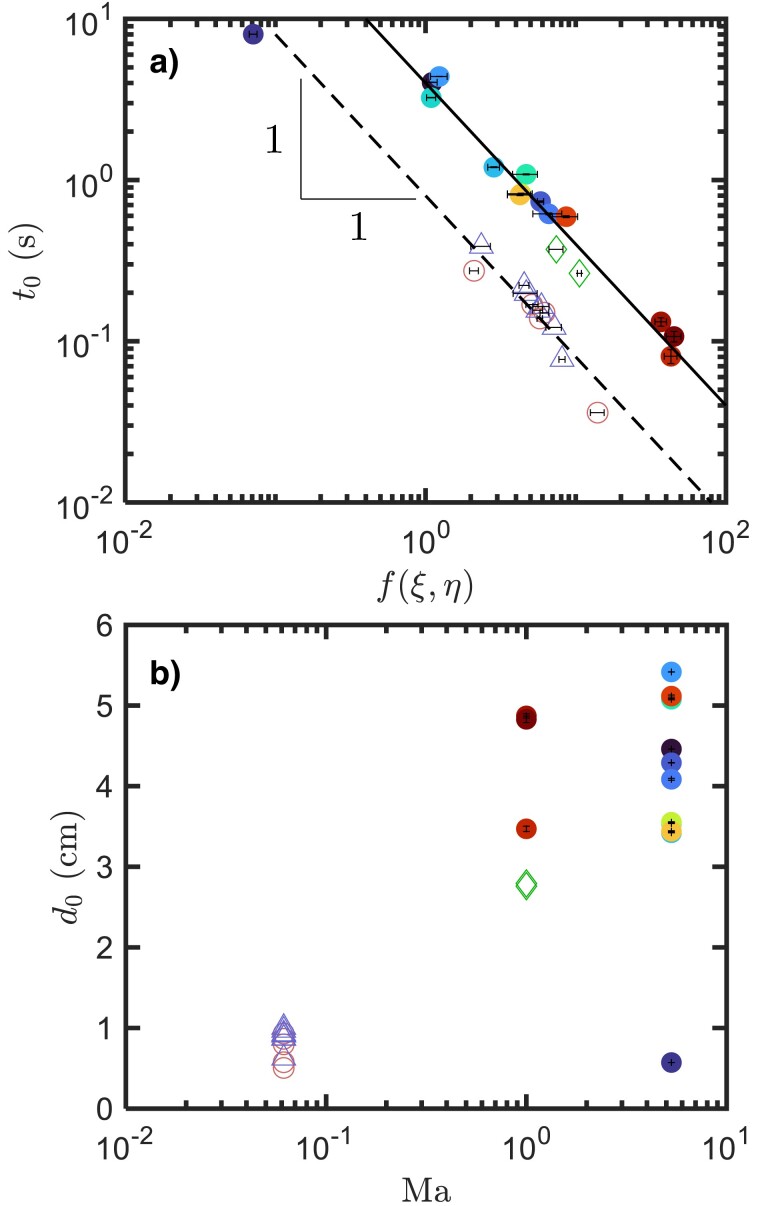
a) The critical time versus the early-stage erosion rate f(ξ,η). The symbols are consistent with Fig. 3. The solid line indicates $t_{0}\propto f(\xi ,\eta {)}^{-1}$. As in 4a, the solid line passes through the sonic and supersonic cases, and the dotted line passes through the subsonic cases. b) The critical depth versus the Mach number.

Most cases seem to follow the 1/3 power law well, which supports the second prediction from the power-balance model. Additional data were acquired from previous works using subsonic (open symbols) (20, 33) and sonic (open diamonds) jets (14) in a terrestrial atmospheric pressure environment. Most of these datasets follow the same transition from the linear to sublinear regimes, as shown in Fig. 3 (open symbols). Using the same approach, values for f(ξ,η) and g(ξ,η) can be extracted from each of these datasets, and the results are also plotted in Fig. 4a. A similar 1/3 scaling is also observed between the two erosion rates, albeit over a smaller range. Note that the sonic data points (diamonds) are close to our results at Ma=1 or 5, whereas the rest of the subsonic data has a much smaller g(ξ,η), indicating that the erosion rate is sensitive to the jet speed. A single supersonic data point falls on the subsonic (dotted) line in Fig. 4a. However, because this data point represents a case with lunar ambient pressure and a low mass flow rate, the jet may become subsonic by the time it impinges on the soil bed. A similar observation and argument may be applied to Fig. 5a. The dotted and solid lines included in Figs. 4a and 5a do not imply that all subsonic cases collapse onto one and all sonic/supersonic cases collapse onto the other, but to show that, for the current parameter space investigated, the difference between supersonic versus subsonic is much larger than the variations within each group separately. In addition, it also emphasizes the importance of jet velocity (Mach number) to the PIC problem.

### Erosion rate

Since the two erosion rates are not independent, we can focus on discussing one erosion rate f(ξ,η), which is a variable that could depend on any combinations of the soil (*ξ*) and gas (*η*) properties varied between experiments. To explore the relationships between f(ξ,η) and ξ/η, a study was performed to determine which parameters f(ξ,η) is the most sensitive to.

No dependence of f(ξ,η) on granular complexity (monodispersed, bidispersed, or tridispersed particles) was observed. The gas jet was affected by various parameters (*η*), including the nozzle height $h/D_{n}$, the mass flow rate $\dot{m}$, and the ambient pressure $P_{\mathrm{amb}}$. Of these parameters, a clear dependence is only evident between f(ξ,η) and $P_{\mathrm{amb}}$. This finding can also be explained by our model in Eq. 1, where the characteristic pressure is a key parameter that determines impingement distribution, and therefore the effective power injected by the jet.

Figure 4b shows the nondimensionalized f(ξ,η) as a function of the nozzle pressure ratio $\mathrm{NPR}=P_{o}/P_{\mathrm{amb}}$, where $P_{o}$ is the jet stagnation pressure. The nondimensionalization of f(ξ,η) is accomplished via the mass flux ratio, which is the ratio between the erosion mass flux ($\rho_{p}f(\xi ,\eta )$) to the gas mass flux ($\rho_{j}V_{j}$). To compare sonic with supersonic cases, $\rho_{j}$ and $V_{j}$ represent the gas density and jet velocity at the nozzle throat where the flow is choked.

The erosion rate f(ξ,η) depends not only on NPR but also on the nozzle height $h/D_{n}$ and the mass flow rate $\dot{m}$. The normalization conducted using $\rho_{j}V_{j}$ already accounted for the difference in the mass flow rate between experiments. To acquire the dependence on $h/D_{n}$, the least square fit of the data shows that $\rho_{p}f(\xi ,\eta )/\rho_{j}V_{j}\propto (h/D_{n}{)}^{\alpha_{h}}$, where $\alpha_{h}=-1.07$. In a model that describes the impingement pressure on a solid surface by a supersonic jet (34), it was suggested that the impingement pressure scales with $(h/D_{n}{)}^{-2}$. By arguing that the velocity scales as the square root of the dynamic pressure, one can arrive at the relationship $f∼(h/D_{n}{)}^{-1}$, which appears to agree with the fitted exponent of $\alpha_{h}=-1.07$. Therefore, the vertical axis in Fig. 4b is $\rho_{p}f(\xi ,\eta )/\rho_{j}V_{j}(h/D_{n}{)}^{\alpha_{h}}$ to remove the nozzle height effect in order to isolate the dependence on NPR, which appears to follow a simple relationship $\rho_{p}f(\xi ,\eta )/\rho_{j}V_{j}(h/D_{n}{)}^{\alpha_{h}}\propto {\text{NPR}}^{-0.77}$. This relationship provides a quantitative way to estimate the erosion rate in environments with different ambient pressures.

### Critical time and depth

As the crater growth transitions from the linear to sublinear regimes, the exact transition time $t_{0}$ and the critical crater depth $d_{c}(t_{0})$ should provide some information about the gas–soil interaction. In addition to using the transition time of $d_{c}(t)/w_{c}(t)$ to determine $t_{0}$ in Fig. 2, $t_{0}$ can also be extracted from $d_{c}$ versus *t* curves; the linear and 1/3 scaling law were fitted to the experimental data, and the intersection between the two fitted lines provides an alternative estimation of $t_{0}$. Here, we chose the difference between these two different methods for calculating $t_{0}$ to represent the uncertainty of $t_{0}$.

Although no obvious relationship between $t_{0}$ and f(ξ,η) was known a priori, $t_{0}$ and f(ξ,η) displayed opposite dependencies on $P_{\mathrm{amb}}$ with similar level of scatter. This motivated us to plot $t_{0}$ against f(ξ,η) directly, which is shown in Fig. 5a. Surprisingly, most points follow the same trend $t_{0}=d_{0}f^{-1}(\xi ,\eta )$ exceptionally well, especially considering how many parameters were varied between different points. However, the value of the fitting parameter $d_{0}$ for the subsonic data approaches a different constant value. Since the Mach number for the subsonic data was held below 0.1, whereas Ma=1 or 5 for the remaining datasets, this suggests that $d_{0}$ seems to be sensitive to the Mach number.

Physically, the fitting parameter $d_{0}$ represents the critical crater depth near the end of the linear growth regime and before the crater widens. In Fig. 5a, the fact that most subsonic datasets collapse on one line and supersonic cases collapse onto another implies that $d_{0}$ is sensitive to Mach number but less so to other parameters varied in these datasets. To show this, $d_{0}$ is extracted from each individual case and plotted against Ma in Fig. 5b. Other than the data point corresponding to the smallest pressure and flow rate, the rest of the data points suggest that the critical depth has a large change from subsonic to sonic.

The two depth growth regimes can be bridged together with one scaling transition model inspired by the generalized Batchelor parameterization (35).


(4) $$d_{c}(t)=f^{1/3}(\xi ,\eta )t^{1/3}{\left[\frac{\;f(\xi ,\zeta )t}{(C_{0}^{-6/\alpha }f^{\alpha }(\xi ,\eta )t^{\alpha }+d_{c}^{\alpha }C_{0}^{-6/\alpha }{)}^{1/\alpha }}\right]}^{2/3}.$$

In this equation, $C_{0}=g(\xi ,\eta )/f^{1/3}(\xi ,\eta )$ is the erosion rate ratio, and *α* controls the “sharpness” of the transition between the linear and 1/3 power law regimes. This formulation satisfies the two limits: for early stage $(t\ll t_{0}),d_{c}(t)=f(\xi ,\eta )t$; for late stage $(t\gg t_{0}),d_{c}(t)=C_{0}f^{1/3}(\xi ,\eta )t^{1/3}$. Furthermore, at $t_{0}$, the two terms in the denominator that controls the weight of each regime should be the same, which provides a constraint, i.e. $C_{0}^{-6/\alpha }d_{c}^{\alpha }=1$, to the value of *α*. The erosion speed ratio $C_{0}$ and the critical depth can be independently extracted from each dataset. For all the cases studied, *α* has a consistent value close to 2 (more precisely, 2.01±0.12 for the current study and 2.10± 0.41 for previous works combined, where the uncertainty is the standard deviation). For the crater depth evolution as shown in Fig. 1d, the dashed lines show the model prediction. The data agrees with the prediction well, further supporting the model.

Finally, the current model indicates that the crater will continue to grow indefinitely following the 1/3 power law because it does not have a mechanism in place for describing the asymptotic crater depth. Such terminal crater growth behavior was studied before by Metzger et al. (12) in subsonic experiments, which showed that, from tens of seconds to about 100 s, the crater growth slows down due to the strong recirculation of gas and particles within the crater. As the focus of the present experiments was on early-time cratering dynamics, the reported phenomena are distinct from this terminal-stage recirculation.

## Conclusion

Granular cratering from the impingement of a high-speed gas jet gives rise to many complex phenomena, from viscous surface erosion to granular fluidization by gas intrusion, and it remains unclear how to describe plume cratering dynamics in a unified framework. Inspired by the scaling laws developed to relate the crater depth and impactor kinetic energy for impact cratering, the power injected by the gas is linked to the kinetic energy gained by the excavated particles per unit time. This model quantitatively captures the temporal evolution of the crater depth for sonic and supersonic gas jets over a wide range of granular compositions (monodispersed to tridispersed), mass flow rates, and nozzle heights.

Within the parameter space investigated, our model suggests that the gas and granular properties do not affect the power laws of the temporal growth of the crater depth; rather, they only affect the initial erosion rate, which can be tied to the late-stage erosion rate and the critical transition time as both depend on its value. We further show that this initial erosion rate seems to scale with the nozzle pressure ratio, as the impinging plume distribution is affected by the ambient pressure.

Landing in a high-vacuum environment, such as the Moon, will result in a larger jet expansion and slower erosion. The scaling laws for crater growth reported in this work have been observed across a range of ambient pressures, from lunar (near-vacuum) to Earth atmospheric pressure. The developed model captures these scaling laws and provides a new framework to guide future landing missions and potentially predict cratering dynamics for a given retrograde firing time.

## Materials and methods

### Experimental setup

An experimental campaign was conducted at NASA Marshall Space Flight Center to obtain cratering data in conditions that simulate the extreme low-pressure environments of the Moon, Mars, and other planetary bodies. The basic setup of the experiment is shown in Fig. 1. A regolith bin was placed underneath a supersonic nozzle. A knife edge on the edge of the regolith bin’s front pane was used to split the crater in half, exposing the crater profile through the transparent front pane.

The entire experiment was conducted inside a $15^{\prime }$ vacuum chamber capable of attaining near-vacuum conditions ($∼10^{-5}$ atmospheres), representative of the conditions present on the lunar surface. Three high-speed cameras were used to capture data: one camera was dedicated to capturing the crater profile evolution, and two cameras were dedicated to capturing trajectories of individual ejecta particles.

In this work, we primarily report results from the high-speed camera that captured the crater profile evolution.

### Particles

The particles tested include monodispersed sand and glass beads (the particle diameter $d_{p}\approx \text{1}25-177$μm), bidispersed sand ($d_{p}\approx \text{1}25-177$μm and $d_{p}\approx \text{4}5-53$μm), and tridispersed sand ($d_{p}\approx \text{2}50-350$μm, $d_{p}\approx \text{1}25-177$μm, and $d_{p}\approx \text{4}5-53$μm). Each constituent of the bidispersed and tridispersed particles was mixed at an equal volume ratio.

### Data

In Fig. 2c, all the datasets were identified by their unique name in the legend. They represent particle, pressure, nozzle height, and mass flow rate. For particles, MD, BD, and TRI stand for monodispersed, bidispersed, tridispersed, respectively. For pressure, Earth, Mars, Moon represent $P_{\mathrm{amb}}\approx 101$ kPa, $P_{\mathrm{amb}}\approx 533$ Pa, and $P_{\mathrm{amb}}\approx 5.33$ Pa. The *h* value represents the normalized nozzle height, e.g. h4 indicates $h/D_{n}=4$. Finally, the ${\dot{m}}_{j}$ value stands for the mass flow rate in grams per second.

The initial crater growth requires resolving small profile changes, which was particularly challenging for some cases in near-lunar conditions coupled with a large nozzle height and/or a low mass flow rate, resulting in very slow erosion dynamics—so slow that the initial crater growth cannot be resolved with the camera. For these cases, the crater depth appeared to be constant for an extended period of time at the beginning of the test, indicating that the small crater growth was beyond the camera resolution and could not be measured accurately.

### Particle velocity

A separate camera was used to capture velocity statistics of individual particles excavated during the PIC process. After an initial transient period of approximately 0.1 s, the mean particle velocity was observed to decay to a plateau.

## Data Availability

All the data supporting this work are available from the corresponding author upon reasonable request.
